# Screening a protein kinase inhibitor library against *Plasmodium falciparum*

**DOI:** 10.1186/s12936-017-2085-4

**Published:** 2017-11-07

**Authors:** Irene Hallyburton, Raffaella Grimaldi, Andrew Woodland, Beatriz Baragaña, Torsten Luksch, Daniel Spinks, Daniel James, Didier Leroy, David Waterson, Alan H. Fairlamb, Paul G. Wyatt, Ian H. Gilbert, Julie A. Frearson

**Affiliations:** 1 Drug Discovery Unit, Division of Biological Chemistry and Drug Discovery, School of Life Sciences, University of Dundee, Dundee, DD1 5EH UK; 2 Medicines for Malaria Venture, Route de Pré-Bois 20, 1215 Geneva 15, Switzerland

## Abstract

**Background:**

Protein kinases have been shown to be key drug targets, especially in the area of oncology. It is of interest to explore the possibilities of protein kinases as a potential target class in *Plasmodium* spp., the causative agents of malaria. However, protein kinase biology in malaria is still being investigated. Therefore, rather than assaying against individual protein kinases, a library of 4731 compounds with protein kinase inhibitor-like scaffolds was screened against the causative parasite, *Plasmodium falciparum*. This approach is more holistic and considers the whole kinome, making it possible to identify compounds that inhibit more than one *P. falciparum* protein kinase, or indeed other malaria targets.

**Results:**

As a result of this screen, 9 active compound series were identified; further validation was carried out on 4 of these series, with 3 being progressed into hits to lead chemistry. The detailed evaluation of one of these series is described.

**Discussion:**

This screening approach proved to be an effective way to identify series for further optimisation against malaria. Compound optimisation was carried out in the absence of knowledge of the molecular target. Some of the series had to be halted for various reasons. Mode of action studies to find the molecular target may be useful when problems prevent further chemical optimisation.

**Conclusions:**

Progressible series were identified through phenotypic screening of a relatively small focused kinase scaffold chemical library.
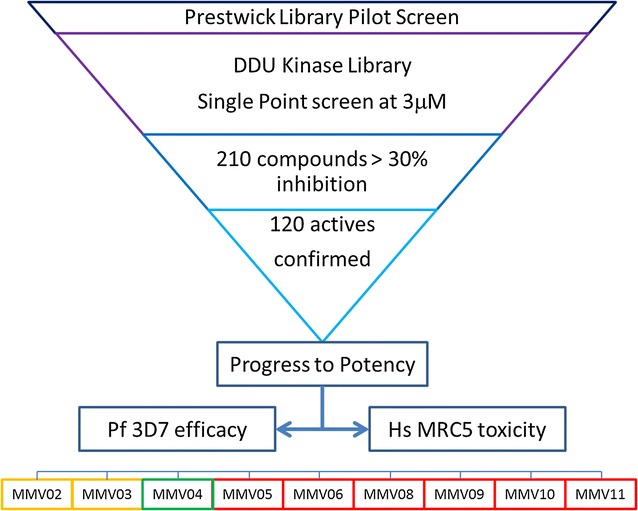

**Electronic supplementary material:**

The online version of this article (10.1186/s12936-017-2085-4) contains supplementary material, which is available to authorized users.

## Background

Resistance of *Plasmodium falciparum* to existing therapy is emerging rapidly [1, 2] and, therefore, much effort is being devoted to discover, develop and deliver new treatments for malaria. The Drug Discovery Unit (DDU) at the University of Dundee has assembled a number of Focused Compound Libraries tailored to certain target classes, such as kinase, protease and phosphatase inhibitors. Protein kinases have been suggested as targets for drug discovery in *Plasmodium* species [3, 4]. The malaria kinome is predicted to contain 85–99 protein kinases [5, 6], of which 65 belong to the eukaryotic protein kinase family and 20 belonging to the FIKK family, unique to the Apicomplexa [6, 7]. The malaria kinome also differs from the human kinome in that it does not contain tyrosine kinases [6]. Malaria kinases typically show only 35–60% sequence identity to their mammalian orthologues suggesting that selective inhibition is possible [8]. Indeed, success has been reported with inhibitors of phosphatidylinositol-4-OH kinase (PI(4)K) [9, 10], albeit this enzyme is a lipid kinase. Although 36 of the 65 eukaryotic protein kinases in *P. falciparum* have been genetically validated as drug targets [11], no inhibitors of these have been developed into clinical candidates to date. However, protein kinase biology in *P. falciparum* is still being investigated. Therefore, rather than assaying against individual protein kinases, it was decided to screen a library of compounds with protein kinase scaffolds in a whole cell assay (phenotypic screening).

Phenotypically screening potential protein kinase inhibitors has the advantage of screening the whole kinome in a more integrated way, and also gives the opportunity to consider a polypharmacology approach by identifying compounds that inhibit more than one *P. falciparum* protein kinase or, indeed, other targets. As part of a pilot study with Medicines for Malaria Venture (MMV), the DDU Kinase Inhibitor library [12], and the commercially available Prestwick Library [13], were screened phenotypically against *P. falciparum* using a DDU optimized SYBR Green assay platform [14]. As a result of further validation, characterization and expansion around key hits, this programme has yielded 9 confirmed scaffolds of interest with sub-micromolar potency.

At the time of the screen, there was no significant published work on 5 of the 9 series, although 2 of the series were part of the ongoing MMV portfolio. Further work was carried out to validate 4 of the series, 3 of which demonstrated sub-micromolar potency against *P. falciparum* with initial SAR and reasonable selectivity against the mammalian MRC5 cell line. The MRC5 cell line is a normal diploid human fibroblast cell line, which is commonly used as a typical counter-screen [15]. These series were chemically tractable, demonstrated excellent selectivity over a panel of mammalian kinases and thus offered excellent opportunities for good start points to enter hit-to-lead programmes.

## Methods

### *Plasmodium falciparum* screening

Cultures of 3D7, a *P. falciparum* chloriquine sensitive reference strain, were maintained in a 5% suspension of A+ human red blood cells (obtained from East of Scotland Blood Transfusion Service, Ninewells Hospital, Dundee, UK) cultured in RPMI 1640 medium (pH 7.3) supplemented with 0.5% Albumax II (Gibco Life Technologies, San Diego, CA, USA), 12 mM sodium bicarbonate, 0.2 mM hypoxanthine, and 20 mg/l gentamicin at 37 °C, in a humidified atmosphere of 1% O_2_, 3% CO_2_ with a balance of nitrogen. Growth inhibition was quantified using a fluorescence assay utilizing the binding of SYBR Green I to double stranded DNA, which emits a fluorescent signal at 528 nm after excitation at 485 nm. The SYBR Green assay system was adapted to maximize robustness and to align with available automation systems. The Prestwick Library then used as a pilot screen to validate the performance of the optimized assay. Mefloquine (potency range 30–60 nM) [16] was used as a drug control to monitor the quality of the assay (Z’ = 0.6–0.8, Signal to background ≥ 3, where Z’ is a measure of the discrimination between the positive and negative controls on a screening plate). Single point screens were carried out, and the potency of compounds of interest was determined in duplicate on two independent occasions. A 96-well [^3^H]-Hypoxanthine incorporation assay [17] was also developed as a secondary assay in order to validate key compounds from each hit series in an orthogonal platform. Compound bioactivity from both assays was expressed as EC_50_, the effective concentration of compound causing 50% parasite death.

### Mammalian toxicity screening

A counter-screen against normal diploid human fibroblasts (MRC-5 pd30, ECACC 84101801) was carried out to exclude non-selective, and toxic compounds as previously published [18]. Cells were plated and incubated overnight to allow them to adhere as monolayers. A working stock of each test compound was transferred to an intermediate 384-well plate and pre-diluted with minimum essential media (MEM). The pre-diluted stocks were then transferred onto the cell monolayers, and the plates were incubated for 68 h. Resazurin, to a final concentration of 50 μM was added to each well, after which plates were incubated for a further 3 h and measured for fluorescence (*λ*_ex_ = 528 nm, *λ*_em_ = 590 nm). A standard reference compound, doxorubicin, was included on all counterscreen plates to monitor the quality of the assay (potency range 200–400 nM).

### In vitro cell assay data analysis

All data were processed using IDBS ActivityBase [19]. Raw data were converted into per cent inhibition through linear regression by setting the high inhibition control as 100% and the no inhibition control as 0%. Quality control criteria for passing plates were as follows: Z’ > 0.5, S:B > 3, %CV_(no inhibition control)_ < 15. The formula used to calculate Z’ is $1 - \frac{3 \times (StDev\_high + StDev\_low)}{ABS(Mean\_high - Mean\_low)}$.

All EC_50_ Curve fitting was undertaken using XLFit version 4.2 using Model 205 with the following 4 parametric equation: $$ y = A + \frac{B - A}{{1 + ({\raise0.7ex\hbox{$C$} \!\mathord{\left/ {\vphantom {C x}}\right.\kern-0pt} \!\lower0.7ex\hbox{$x$}})^{D} }}, $$ where A = % inhibition at bottom, B = % inhibition at top, C = EC_50_, D = slope, *x* = inhibitor concentration and y = % inhibition. If the curve did not reach 100% of inhibition, B was fixed to 100 when at least 50% of inhibition was reached.

### Compound library

Protein kinases have been suggested as potential drug targets in malaria [3, 7]. However, rather than screen each individual kinase, a more rapid approach would be to screen the malaria parasite phenotypically. This has a number of advantages: it is not necessary to clone and overexpress each kinase and develop an individual assay; the protein kinase is in its relevant state of activation with the relevant substrates present; this only selects cell penetrant compounds and compounds that have a lethal effect; it would detect compounds that were acting on multiple proteins. The disadvantages of this approach include the unknown identity of the target(s), which could make optimization of hits problematic, particularly if there were pharmacokinetic or toxicological issues.

The Dundee library of protein kinase scaffolds was assembled as described in Brenk et al. [12]. The aim of this library was to find chemical start points for drug discovery programmes, so the molecules were deliberately kept low molecular weight, giving room for optimisation. In brief, a review of the literature and patents was carried out by industry experienced chemists to identify scaffolds that bind in the adenine pocket of all protein kinases, to the so-called hinge region. Allosteric inhibitors were not considered. The hinge region is part of the backbone that forms a hydrogen bonding network with the adenine moiety of ATP. Decoration of these scaffolds can achieve varying degrees of selectivity for particular kinases. These scaffolds were then searched for using an in silico database of 2.6 million discrete compounds compiled from the catalogues of 26 suppliers. The initial selections were further screened to eliminate non-lead like compounds, using over 100 filters based on chemically reactive groups and toxicophores. This process identified in excess of 150 scaffolds with at least one commercially available example. To limit the total number of compounds in the screening collection, a limit of the most diverse 50 examples of each scaffold was set using Tanimoto Indices to assess similarities. At the time of the screen this library contained 4731 individual compounds.

### Hit discovery

The 4731 compounds from the DDU curated kinase library were screened against *P. falciparum* at a single concentration of 3 μM using a fluorescence-based assay. Compounds with a percentage inhibition (PI) > 30 were retested in duplicate and 120 hits were progressed to potency determination against both *P. falciparum* and human MRC5 cells to assess toxicity. Potency values were established using a 10-point, half-log serial dilution of each compound from a top concentration of 15 µM. Two independent assays were run. Screening data statistics were comparable with those obtained in the pilot screen.

To further establish all discovered hits, structural identity and purity of all compounds progressed to potency was confirmed by LC/MS. Fresh material was sourced by resupply or resynthesis to confirm activity in the original assay and in an orthogonal (hypoxanthine incorporation) assay.

### Kinase profiling

The compounds were cherry picked from library material and tested at 10 µM. This was carried out at the International Centre for Kinase Profiling, University of Dundee [20], where the principal assay method utilized is a radioactive filter-binding assay using ^33^P ATP. At the time of screening, there were 76 protein kinases in the panel. Whilst this represents a relatively small proportion (~ 15%) of the mammalian kinome (518 predicted protein kinases in humans [21]), it should give an indication as to whether the compound is selective or promiscuous. The profiled compounds were checked for purity and molecular identity by LCMS.

### LCMS QA

LCMS analysis of the 120 compounds progressed into EC_50_ determination was conducted on an Agilent technologies 1200 series LC/Agilent technologies 6130 quadrapole LC/MS, eluting with 20–90% MeCN in 0.1% aqueous formic acid. 96 of the 120 compounds had > 85% purity by diode array and confirmation of the expected mass to give an 80% pass rate.

## Results

### Pilot screen

**Fig. 1:**
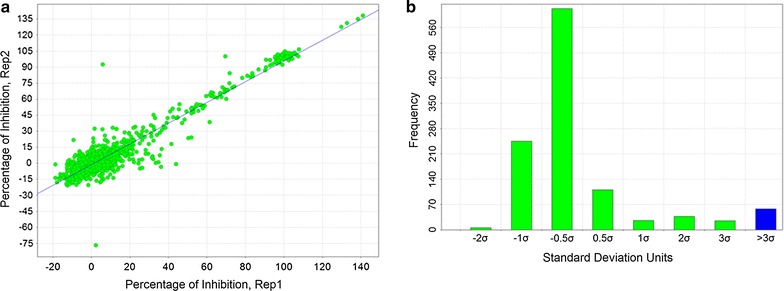
Prestwick library screen (concentration approximately 5 μM). **a** Correlation plot of the percentage of inhibition of the two replicate values. **b** Frequency distribution plot expressed in terms of standard deviation units (s) from the median value of the controls. In blue are highlighted the 58 putative hits with percentage of inhibition higher than 3 s units

In order to validate the optimized assay, the commercially available Prestwick Library (1120 compounds) was screened in duplicate at single concentration. A number of compounds with known anti-malarial properties were identified from this pilot screen, and results compare favourably with published data [22] (Fig. 1, Table 1 and Additional file 1: Table S1 for full list of the hits), thus indicating that the DDU optimized assay was fit for purpose. The final assay was demonstrated to be robust (Z’ = 0.84 ± 0.01), and reproducible (mefloquine) EC_50_ = 48 ± 4 nM (n = 24).

**Table 1:** Percentage inhibition of the growth *of Plasmodium falciparum* of known anti-malarials in the Prestwick Library

Name	Screening conc. (mM)	Percentage inhibition
Chloroquine diphosphate	0.004	104 ± 1
Pyrimethamine	0.008	101 ± 2
Artemisinin	0.007	100
Primaquine diphosphate	0.004	100 ± 4
Hydroquinine hydrobromide hydrate	0.005	99 ± 2
Atovaquone	0.005	99 ± 2
Mefloquine hydrochloride	0.005	97.4 ± 0.4
Proguanil hydrochloride	0.007	49 ± 4

### Primary screen

**Fig. 2:**
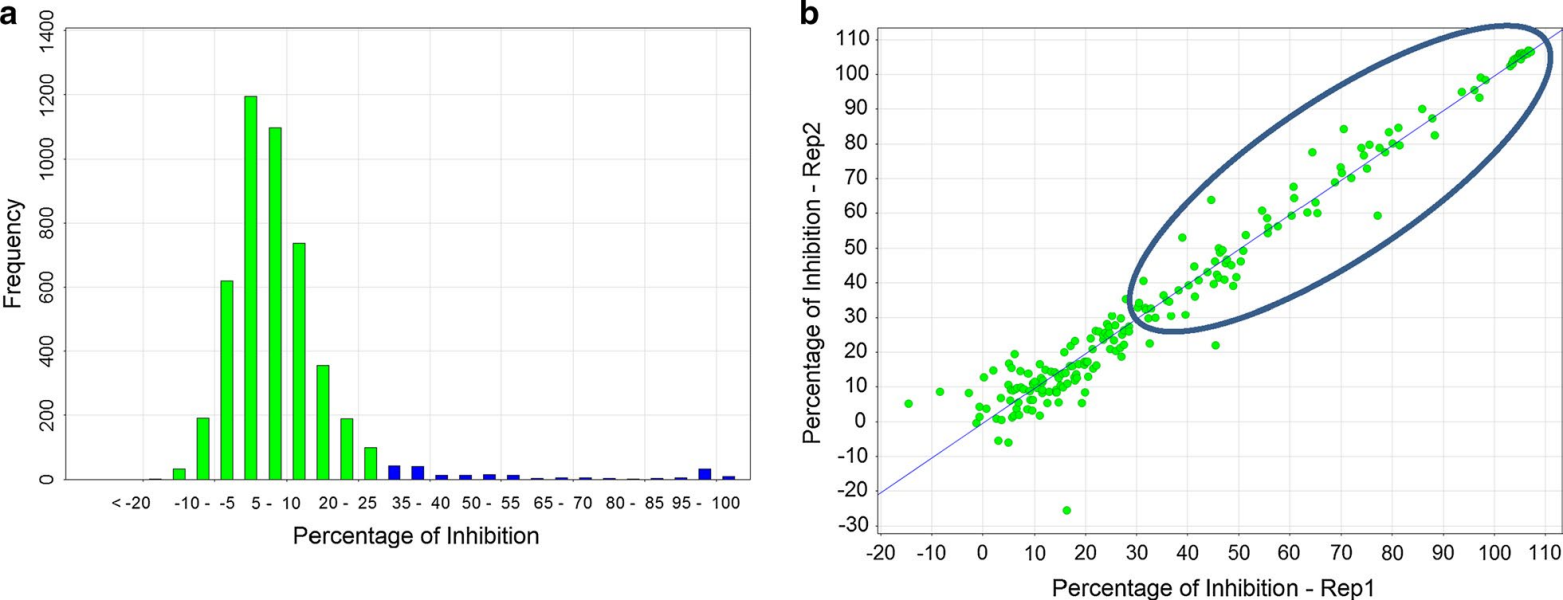
Kinase Set Screen. **a** Distribution of percentage of inhibition. In blue are highlighted the hits with percentage of inhibition higher than 30%. **b** Correlation plot between retest replicates. Reconfirmed hits are highlighted in the blue circle

The frequency distribution plot of percentage inhibition for the primary screen of the focused kinase library against *P. falciparum* is shown in Fig. 2a, illustrating a classical normal distribution. A cut-off of 30% inhibition was chosen to define an initial hit (3 standard deviations away from the averaged high controls). Table 2 summarizes the numerical results from the primary screen.

**Table 2:** Summary of primary screen

Number of compounds screened	4371
Hit identification cut-off (3 × SD from mean of 0% inhib. controls)	30%
Number of putative hits cherry picked	210

**Fig. 3:**
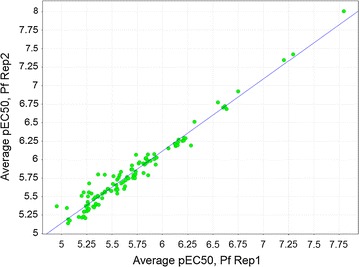
EC50 Correlation of potency replicates in *Plasmodium falciparum*

The 210 putative hits identified from the kinase set primary screen were cherry picked and retested in a duplicate single point screen (3 μM). Figure 3 shows the excellent correlation between the two replicate retest data points. For those compounds returning > 50% inhibition in the primary assay the confirmation rate to retest was 68%. 120 compounds with a percentage inhibition of > 80% were selected for progression to potency testing. Ten-point dose response curves were generated and assayed in both *P. falciparum* and mammalian MRC5 (human lung fibroblasts) proliferation assays. A range of potencies (Table 3) were identified against *P. falciparum*, with the majority of compounds being inactive in the MRC5 counter-screen with an EC_50_ > 15 μM.

**Table 3:** Kinase set potency ranges

EC_50_ (μM)	Number of compounds
EC_50_ < 1	21
1 < EC_50_ ≤ 3	51
3 < EC_50_ ≤ 10	43

### Hit validation

#### Series classification

Hit compounds that had EC_50_ values of less than 1 μM were grouped into compound series using in silico core fragment identification. The sub-micromolar hits identified from screening the DDU focused kinase library were grouped into 9 compound series (summarized in Table 4). The core structures common to hits in each compound series are highlighted in red and the most active example identified in the screen exemplified with the substituents illustrated in black. Most of the compound series were apparent singletons with a large drop in activity to the second most active member of the compound series.

**Table 4:** Sub-micromolar hits identified from screening the DDU focused kinase library were grouped into 9 compound series

Series ID			
	MMV02	MMV03	MMV04
Compound	A0002	A0003	A0004
EC_50_ *P. falciparum* (μM)	0.2	0.6	0.7
EC_50_ MRC5 cells (μM)	6	> 15	> 15
cLogP	4	3.2	2.9
cLogD	4	3.2	2.9
TPSA (Å^2^)	51	60	68
MW	342	346	405
Heavy Atoms	26	25	26
No. of examples < 1 μM	1	1	2
Validated hit series	Yes	Yes	Yes

Series ID			
	MMV05	MMV06	MMV08
Compound	A0005	A0006	A0008
EC_50_ *P. falciparum* (μM)	0.01	0.04	0.6
EC_50_ MRC5 cells (μM)	1	> 15	0.02
cLogP	5.4	4.2	5.7
cLogD	5.4	4.2	5.7
TPSA (Å^2^)	71	57	70
MW	407	355	382
Heavy Atoms	31	27	27
No. of examples < 1μM	1	1	1
Validated hit series	No	No	No

Series ID			
	MMV09	MMV10	MMV11
Compound	A0009	A0010	A0011
EC_50_ *P. falciparum* (μM)	0.2	0.6	0.6
EC_50_ MRC5 cells (μM)	> 15	> 15	6
cLogP	2.6	2.4	4.3
cLogD	2.6	2.4	3.9
TPSA (Å^2^)	55	67	58
MW	348	348	482
Heavy Atoms	24	23	35
No. of examples < 1μM	1	1	1
Validated hit series	No	No	No

In order to avoid overlapping the work conducted in this programme with other MMV collaborations, the series we subsequently focused upon in hit expansion were MMV02 [23], MMV03 and MMV04 [24, 25], as previously published.

#### Orthogonal testing with [^3^H]-hypoxanthine incorporation assay

The traditionally used [^3^H]-hypoxanthine incorporation assay is labour intensive, low throughput and requires the use of radio-labelled hypoxanthine. Therefore, it was used only to test and confirm key series representative hits that had been identified in the SYBR Green assay. The assay as developed yielded acceptable performance metrics (Z’ = 0.68 ± 0.05 and S/B > 300). In addition, mefloquine returned EC_50_ values of 7–10 nM in this assay, which were consistent with those reported by the SYBR Green screening platform. Table 5 demonstrates that all 9 MMV series were validated in this orthogonal platform and that the hits all returned potency values within acceptable ranges of the SYBR Green assay (ratios between 1 and 4 observed).

**Table 5:** Comparison of compound EC_50_ values obtained by fluorescent (SYBR Green), and radiometric ([^3^H]-hypoxanthine) assay platforms

MMV series	Compound	EC_50_, mM		Ratio fluorescent vs radiometric
		Fluorescent C.I. 95%, n = 4	Radiometric C.I. 95%, n = 2	
MMV02	**A0002**	0.23 ± 0.04	0.06 ± 0.07	4
MMV03	**A0003**	0.59 ± 0.12	0.15 ± 0.25	4
MMV04	**A0004**	0.70 ± 0.07	0.41 ± 0.47	2
MMV05	**A0005**	0.01 ± 0.00	0.01 ± 0.00	1
MMV06	**A0006**	0.04 ± 0.01	0.04 ± 0.01	1
MMV08	**A0008**	0.56 ± 0.09	0.30 ± 0.10	2
MMV09	**A0009**	0.23 ± 0.09	0.15 ± 0.09	2
MMV10	**A0010**	0.55 ± 0.08	0.35 ± 0.13	2
MMV11	**A0011**	0.64 ± 0.10	0.19 ± 0.02	3

#### Kinase profiling

Ten compounds across the 9 MMV series were screened in vitro against a panel of 76 mammalian kinases by the Division of Signal Transduction Therapy (DSTT) at The University of Dundee for selectivity assessment.

The selected compounds, demonstrated excellent selectivity with respect to the 76 kinases tested (Additional file 1: Table S2). The lack of cross-activity against mammalian kinases means that either the identified series are selectively inhibiting plasmodium protein kinase(s) or they have a different, non-kinase based, mode of action. Future assessment of these series against a *Plasmodium* kinase panel will help clarify the mode of action.

### Medicinal chemistry evaluation

#### Further discussion on the preliminary structure activity relationships is included in Additional file 1

The series not already under development by MMV at the time of screening were MMV02, MMV03, MMV04 and MMV10. As such, efforts focused on establishing some early structure activity relationships for the 4 unexplored series.

**Fig. 4:**
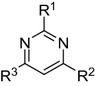
The MMV 02 Scaffold

MMV02 consists of a 1,3,5-tri-substituted pyrimidine core (Fig. 4). The work on this has recently been published [23]. The active MMV02 hit **A002** possessed a 4-aminopyridine substituent as the R^2^ group which was unique within the compound set screened; it was hypothesized that the pyridine nitrogen may be forming a critical hydrogen bond with the molecular target. A further 23 examples of this scaffold were purchased and an additional seven examples tested using stocks of compounds in the DDU general compound set. Interestingly the five most active examples all contained a 4-aminopyridyl group at the R^2^ position (for instance **A0021**). However, **A0022** was only tenfold less active than **A0002**. **A0022** possesses a 4-trifluoromethoxyaniline instead of the 4-aminopyridine of compound **A0002** (Table 6). The ether oxygen of 4-trifluoromethoxyaniline group is a very poor hydrogen bonding unit, which is unlikely to be able to form a hydrogen bond to the hinge NH of a kinase [26, 27]. This suggests that kinase inhibition is unlikely to be responsible for the mode of action for this series.

**Table 6:**
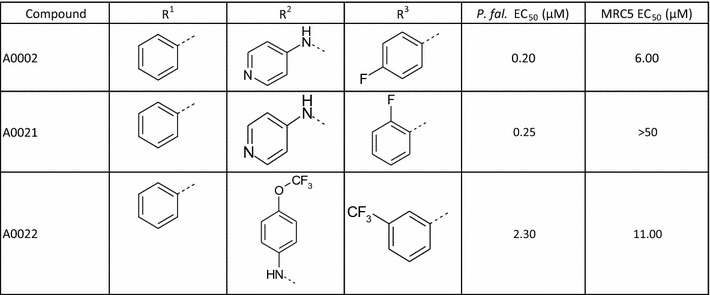
Key MMV02 SAR

Data reported previously for **A0021** [23]

**Fig. 5:**
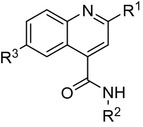
MMV04 Scaffold

MMV04 is a 2,6-disubstituted quinoline-4-carboxamide (Fig. 5). Work describing this series has been published [24, 25]. The screen identified two sub-micromolar hits, **A0004** and **A0031**. In the hit expansion programme, 84 analogues were purchased. Modifying the R^1^ group had a significant effect on activity with 4-tolyl **A0031** fivefold more active than **A0004**, (Table 7). The 3-pyridyl amide in **A0004** (R^2^ position) could be replaced with ethyl phenyl-4-carboxylate **A0024** with no significant effect on activity. The R^2^ region tolerated large changes to ring electronics and steric bulk (**A00410**), suggesting that this region is not critical for maintenance of activity. Examples **A0004** and **A00421** demonstrated that the bromine (R^3^) can be replaced with the smaller chlorine group with no loss of activity and a reduction in the MW of 55.

**Table 7:**
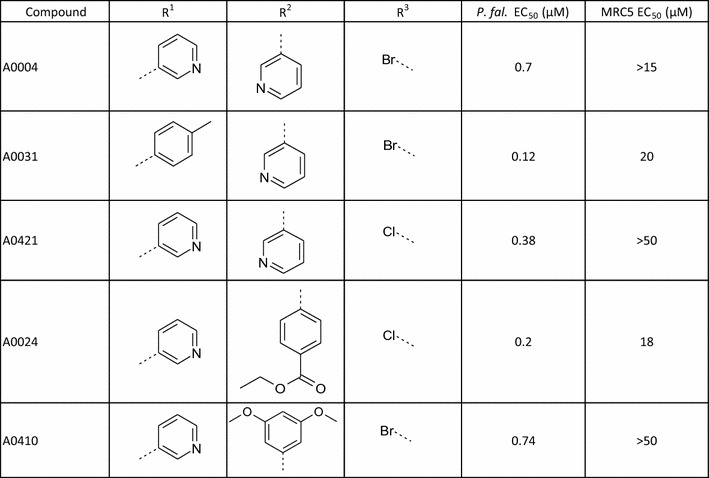
MMV04 SAR

Data reported previously for A0031 [24, 25]

**Fig. 6:**
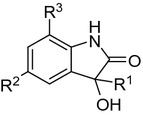
MMV10 Scaffold

MMV10 was identified as a singleton from the screen. The series was defined as a 3,5,7-trisubstituted 3-hydroxy-indolin-2-one (Fig. 6). The hit compound **A0010** possesses a 2-methylimidazo[1,2-a]pyridin-3-yl which could be responsible for a critical hydrogen bond, Table 4. The initial hit was no longer commercially available. An additional 15 examples were purchased, none of which were progressed into EC_50_ determination. MMV10 could not be validated as a hit series nor has the original hit been reconfirmed. In order for MMV10 to be progressed further, the original hit would need to be re-synthesized along with some close analogues.

Following hit validation, the hit to lead and lead optimisation medicinal chemistry programmes for series MMV02 and MMV04 have been published elsewhere [23–25].

#### MMV03 validation and hit expansion

**Fig. 7:**
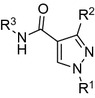
MMV03 Scaffold

MMV03 has a 1,3-disubstituted pyrazole-4-carboxamide core (Fig. 7). Only one sub-micromolar hit, **A0003**, was identified following screening, Table 8. A further 62 analogues were sourced and tested single point at 10 μM; of these, 11 compounds active in single point were progressed into EC_50_ determination returning EC_50_ values ranging from 0.32 to 7.2 µM. **A0003** remained the most active example in this series. All examples with EC_50_ values less than 10 µM have a pyridine-like (H-bond acceptor) nitrogen containing group on the amide, though the nitrogen can be orientated in the 2, 3 or 4 position for instance compounds **A0003**, **A0032**, and **A0033**.

**Table 8:**
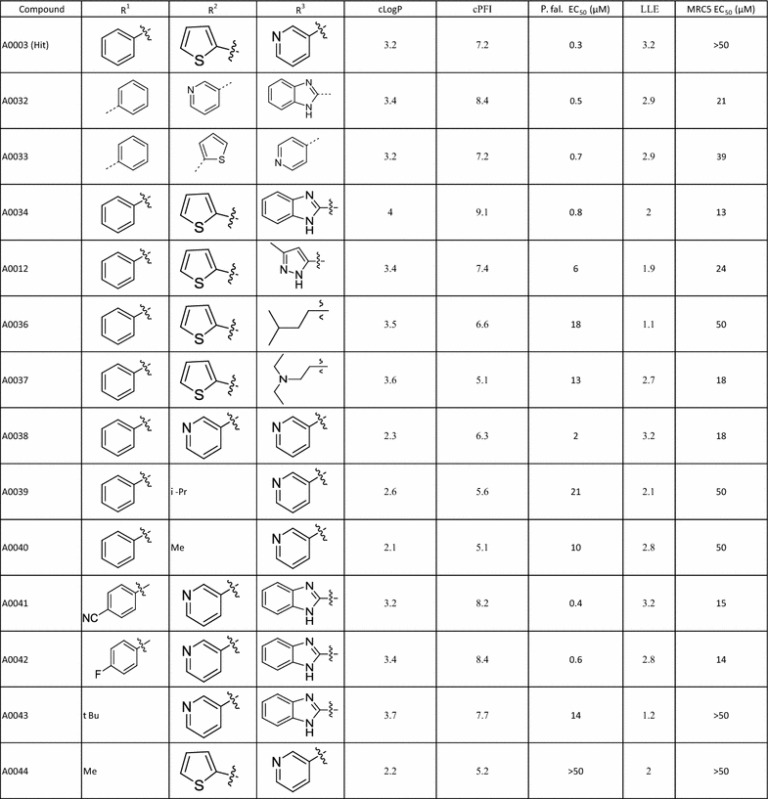
MMV03 SAR

The most active compound of the original hit series displayed moderate activity in vitro and good selectivity index (> 100-fold) against human MRC-5 cell line (Table 8). However, the ligand-lipophilicity efficiency (LLE or LiPE) [28] was low and the calculated property forecast index (cPFI) [29], an indicator of compound development potential, was unfavourably high. The aim of the initial hit optimization programme was to improve both the physicochemical properties and potency of this series (with an aim of *Pf* EC_50_ (3D7) < 0.1 μM).

Initial modifications were directed towards improving the physicochemical properties of the compounds, particularly reducing lipophilicity and the number of aromatic rings and therefore reducing cPFI. Large numbers of aromatic rings are associated with poor compound development potential [30, 31]. The substituents R^1^, R^2^ and R^3^ (see Fig. 7 and Table 8 for definitions of the R groups) were aromatic rings in the initial hit.

First, the amide substituent (R^3^) was extensively explored. All compounds with activity below 1 μM have either a pyridine or a 2-benzoimidazole moiety at the R^3^ position. Five membered ring heterocycles, basic and alkyl substituents, with improved cPFI, at the R^3^ position led to a drop in potency. The R^2^ substituent could tolerate thiophene, pyridine and phenyl rings but compounds with an alkyl substituent, methyl or isopropyl, showed a significantly drop in activity (EC_50_ > 10 μM). Finally, the aromatic ring at R^1^ tolerated an electron withdrawing (CN) and donating (F) groups. However, replacement of the aromatic ring with an alkyl group (*tert*-butyl and methyl) was not tolerated.

Therefore, none of 3 aromatic substituents on MMV03 scaffold could be replaced by an alkyl group making the optimization of physicochemical properties problematic. Additionally, the potency and LLE of the hit has not been improved. As a result work on this series was discontinued.

## Conclusion

A *P. falciparum* SYBR Green assay platform has been adapted to robotics available in the University of Dundee DDU, demonstrating robust, reproducible output with performance and results to match the traditionally used [^3^H]-hypoxanthine incorporation assay. Phenotypic screening of an in-house protein kinase inhibitor library identified 9 chemical scaffolds. All series were confirmed using a combination of LCMS QA of the DMSO library stocks and hit repurchasing where possible. Furthermore, 4 of the 9 compound series (MMV10, MMV02, MMV03, MMV04) were further validated as hit series through hit expansion with commercially available compounds. Of these validated series, 3 compound series showed some early SAR, were chemically tractable, and had not previously been developed as potential anti-malarial drugs. These start points were suitable for progression into hit to lead programs for novel anti-malarial therapy.

Whilst the screening library was based on scaffolds designed to bind to the hinge region of eukaryotic protein kinases, there is no evidence that the compounds actually function in this manner. Indeed, the only series for which information is available is the MMV04 series, where the preclinical candidate targets eukaryotic elongation factor 2 (eEF2) involved in protein synthesis [24]. Further work will be required to elucidate the mode of action of the other hit series using approaches such as protein kinase profiling, whole genome sequencing of resistant mutants or chemical proteomics.

## Additional file

**Additional file 1.** Supplementary information.
