# Supplementary material for: Screening a protein kinase inhibitor library against Plasmodium falciparum
Source: Malar J. 2017 Nov 7;16:446. doi: 10.1186/s12936-017-2085-4 (PMC5678585; doi:10.1186/s12936-017-2085-4)
Supplement: Supplementary file 1 — Additional file 1. Supplementary information. [file 12936_2017_2085_MOESM1_ESM.pdf]

SUPPLEMENTARY INFORMATION

Screening a Protein Kinase Inhibitor Library against *Plasmodium falciparum*

Irene Hallyburton,[a] Raffaella Grimaldi,[a] Andrew Woodland,[a] Beatriz Baragaña,[a] Torsten Luksch,[a] Daniel Spinks,[a] Daniel James,[a] Didier Leroy,[b] David Waterson,[b] Alan H. Fairlamb,[a] Paul G. Wyatt,[a] Ian H. Gilbert,[a,*] Julie A. Frearson.[a]

a. Drug Discovery Unit, Division of Biological Chemistry and Drug Discovery, School of Life Sciences, University of Dundee, Dundee, DD1 5EH, UK.

b. Medicines for Malaria Venture, 20, Route de Pré-Bois, 1215 Geneva 15, Switzerland

Tables and Figures

Table S1. Inhibitors with known anti-malarial activity identified in the pilot screen

Table S2 – Percentage activity of the panel of mammalian protein kinases when treated with hit compounds at a concentration of 10 μM.

Figure S1 – MMV05 Scaffold

Figure S2 – MMV06 Scaffold

Figure S3 – MMV08 Scaffold

Figure S4 – MMV09 Scaffold

Figure S5 – MMV11 Scaffold

Figure S6 - General synthetic route.

Name	Concentration, mM	Percentage of Inhibition
Mitoxantrone dihydrochloride	0.004	140 ± 2
Doxorubicin hydrochloride	0.003	137 ± 2
Daunorubicin hydrochloride	0.004	132 ± 1
Pyrvinium pamoate	0.001	107 ± 1
Demecarium bromide	0.003	104 ± 2
**Chloroquine diphosphate**	0.004	104 ± 1
Emetine dihydrochloride	0.004	103 ± 2
Cycloheximide	0.007	103.1 ± 0.1
Benzethonium chloride	0.004	102.5 ± 0.7
Anisomycin	0.008	102 ± 2
Cephaeline dihydrochloride heptahydrate	0.003	101 ± 3
Cinchonine	0.007	101 ± 5
**Pyrimethamine**	0.008	101 ± 2
Methotrexate	0.004	100.9 ± 0.7
Suloctidil	0.006	101 ± 1
(-)-Cinchonidine	0.007	100 ± 1
Pentamidine isethionate	0.003	100 ± 4
Quinacrine dihydrochloride dihydrate	0.004	100± 2
Quinidine hydrochloride monohydrate	0.005	100 ± 2
**Artemisinin**	0.007	99.5 ± 0.1
**Primaquine diphosphate**	0.004	100 ± 4
**Hydroquinine hydrobromide hydrate**	0.005	99 ± 2
**Atovaquone**	0.005	99 ± 2
Berberine chloride	0.005	99 ± 2
Propafenone hydrochloride	0.005	99 ± 1
Chlorhexidine	0.004	98 ± 1
**Mefloquine hydrochloride**	0.005	97.4 ± 0.4
Astemizole	0.004	97.3 ± 0.8
Puromycin dihydrochloride	0.004	96 ± 5
Verteporfin	0.003	95 ± 2
Doxazosin mesylate	0.004	84.0 ± 0.6
(d,l)-Tetrahydroberberine	0.006	83 ± 2
Cyclosporin A	0.002	81.4 ± 0.6
Thonzonium bromide	0.003	70 ± 1
Triamterene	0.008	69 ± 1
Bufexamac	0.009	69 ± 2
Paclitaxel	0.002	65 ± 2
Decamethonium bromide	0.005	64 ± 3
Mycophenolic acid	0.006	63 ± 3
Metergoline	0.005	56 ± 2
Bephenium hydroxynaphthoate	0.005	55.1 ± 0.5
Clobetasol propionate	0.004	50 ± 16
**Proguanil hydrochloride**	0.007	49 ± 4

Table S1. Inhibitors with known anti-malarial activity identified in the pilot screen (Prestwick Library). Antimalarial drugs are shown in bold.

MMV Series	MMV02	MMV03		MMV04	MMV05	MMV06	MMV08	MMV09	MMV10	MMV11
**Compound**	A0002	A0012	A0003	A0004	A0005	A0006	A0008	A0009	A0010	A0011
**MKK1**	36	109	93	99	84	57	72	94	79	90
**ERK1**	97	92	101	84	91	108	113	97	92	108
**ERK2**	124	89	96	108	116	110	106	103	101	110
**JNK1**	93	101	103	97	112	110	104	96	101	105
**JNK2**	86	97	117	126	126	117	111	125	100	129
**p38a MAPK**	66	74	68	90	80	86	97	84	74	90
**p38b MAPK**	63	67	78	95	98	100	112	100	87	95
**p38g MAPK**	88	103	95	102	100	100	111	101	93	113
**p38d MAPK**	85	88	97	101	94	97	111	89	83	89
**ERK8**	86	78	90	88	104	57	74	90	97	55
**RSK1**	89	89	82	96	109	111	66	87	101	42
**RSK2**	66	77	114	107	97	116	104	113	87	48
**PDK1**	105	101	98	93	96	106	112	100	91	121
**PKBa**	37	102	98	97	83	117	110	93	99	71
**PKBb**	58	10	112	100	87	109	101	105	116	105
**SGK1**	83	88	88	98	113	100	89	87	84	86
**S6K1**	95	124	98	112	100	120	115	96	105	125
**PKA**	96	104	96	97	77	95	90	84	94	96
**ROCK2**	93	103	93	111	98	100	94	96	112	101
**PRK2**	90	101	94	101	92	98	106	93	92	76
**PKCa**	120	69	60	86	87	115	75	88	89	88
**PKCZ**	75	91	86	88	88	82	93	92	89	103
**PKD1**	56	128	110	100	101	112	96	100	95	102
**MSK1**	56	112	109	104	101	100	113	89	110	84
**MNK1**	44	90	93	96	98	113	85	103	93	76
**MNK2**	44	93	97	101	104	88	52	87	100	81
**MAPKAP-K2**	108	104	108	97	128	102	92	93	89	126
**PRAK**	98	89	86	101	89	104	78	94	100	107
**CAMKKb**	92	88	89	103	92	71	75	74	100	89
**CAMK1**	7	76	99	109	33	96	76	95	101	59
**SmMLCK**	22	72	219	95	39	106	31	92	112	73
**PHK**	98	105	97	118	122	110	68	106	112	73
**CHK1**	80	97	99	84	109	113	99	95	88	102
**CHK2**	61	64	92	94	93	48	72	94	82	107
**GSK3b**	92	97	106	96	58	100	102	105	95	120

**CDK2-Cyclin A**	97	89	89	96	103	95	99	96	96	86
**PLK1 (Okadaic Acid)**	108	87	89	108	115	109	102	84	99	120
**Aurora B**	72	76	89	105	109	57	81	85	95	23
**AMPK**	47	86	102	107	94	104	99	94	117	88
**MARK3**	98	105	104	111	110	107	114	103	101	110
**BRSK2**	88	73	87	100	95	100	109	97	79	138
**MELK**	97	105	102	86	79	90	114	108	106	77
**CK1**	105	121	90	97	100	121	40	100	110	107
**CK2**	103	97	101	104	99	97	93	91	116	109
**DYRK1A**	86	81	79	92	96	94	62	93	85	117
**DYRK2**	90	88	114	104	92	93	71	101	99	111
**DYRK3**	89	99	91	92	91	92	109	87	83	113
**NEK2a**	91	101	93	91	89	87	84	87	93	79
**NEK6**	101	94	100	88	103	109	103	106	91	108
**IKKb**	137	200	196	101	110	99	103	198	208	125
**PIM1**	94	103	102	93	113	104	100	101	105	97
**PIM2**	100	96	103	90	83	104	118	97	86	123
**PIM3**	89	62	35	22	76	90	88	97	78	82
**SRPK1**	88	81	97	106	102	124	95	88	96	116
**MST2**	97	92	96	103	85	92	108	92	98	92
**EF2K**	83	105	107	106	99	125	114	93	109	101
**HIPK2**	86	93	99	93	86	106	111	90	98	102
**PAK4**	45	76	94	105	77	53	77	85	99	57
**PAK5**	87	77	76	93	140	82	97	92	96	87
**PAK6**	100	109	111	111	115	100	109	89	105	117
**Src**	103	81	90	106	103	64	92	95	95	85
**Lck**	53	116	80	92	91	65	90	109	87	39
**CSK**	98	112	105	88	112	99	99	94	89	112
**FGF-R1**	103	58	75	128	100	97	115	100	91	73
**IRR**	65	70	77	80	86	90	67	90	56	99
**EPH A2**	96	96	91	108	96	107	98	115	94	61
**MST4**	77	86	83	105	69	90	106	93	90	88
**SYK**	108	84	85	102	132	114	99	95	91	103
**YES1**	84	83	61	101	95	45	76	80	81	53
**IGF-1R**	107	90	102	109	128	116	116	110	109	97
**VEG-FR**	91	53	98	97	99	81	77	100	99	87
**BTK**	85	87	86	100	92	77	100	90	80	51
**IR-HIS**	78	97	66	78	92	92	47	76	86	76
**EPH-B3**	88	81	71	108	97	102	95	78	76	103
**TBK1 (du12569)**	102	104	97	101	104	112	108	109	94	123
**IKKe(14231)**	88	98	88	98	98	92	103	103	95	101

Table S2 – Percentage activity of the panel of mammalian (mainly human) protein kinases when treated with hit compounds at a concentration of 10 μM.

Supplementary information: **Medicinal Chemistry Evaluation**

MMV05

MMV05 is a 3,6-disubstituted 1H-pyrazolo[3,4-b]pyridine (Supplementary Figure 1). Only one compound A0005 was identified in the initial screening campaign, this compound could not be repurchased and was already under development by MMV so no further work was conducted on this series. The hit compound A0005 is 10 nM *vs*. *P. falciparum* and showed a 100 fold selectivity window over MRC5 cells. A0005 was tested vs. the 76 kinases in the DSTT panel, inhibiting only CAMK1 and SmMLCK by 67% and 61% respectively at 10 µM. The presence of a large number of metabolically labile groups would be of concern if this series was to be progressed. Table 3 in main body of the paper contains a summary of A0005 and its physical properties as generated by Stardrop.

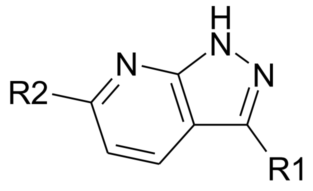

Figure S1 – MMV05 Scaffold

MMV06

MMV06 is a 2,6-disubstituted pyrimidine (Supplementary Figure 2). Only one example of MMV06 was identified in the initial screening campaign, A0006. A0006 is very active against *P. falciparum* and >350 fold selective over MRC5 cells. A0006 was tested in the DSTT kinase panel inhibiting CHK2 and YES1 by 52% and 55% respectively at 10 µM. A0006 is no longer commercially available so could not be reconfirmed and since MMV06 was under development by MMV, no further work was conducted on this series. Table 3 (main body of the paper) contains a summary of A0006 and its physical properties as generated by Stardrop.

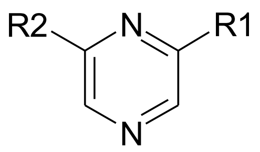

Figure S2 – MMV06 Scaffold

MMV08

MMV08 consists of a 3,8-disubstituted imidazo[1,2-a]pyrazine core (Supplementary Figure 3). Only one example of MMV08 was identified in the primary screen. The hit A0008 has a low nanomolar EC_{50} against MRC5 cells but is over 20 fold less active against *P. falciparum*. Interestingly the MRC5 toxicity is not reflected in the kinase panel results, with only SmMLCK, CK1 and IR-HIS inhibited by between 50-70% at 10 µM. The MMV08 hit could not be repurchased and as MMV was already developing the series we did not progress it into hit expansion. Table 3 (main body of the paper) contains a summary of A0008 and its physical properties as generated by Stardrop.

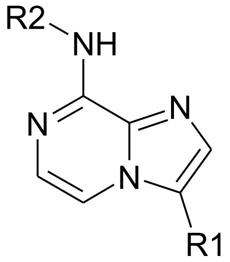

Figure S3 – MMV08 Scaffold

MMV09

MMV09 is a 1,4'-disubstituted 1-(thiazol-2-yl)ethanol (Supplementary Figure 4). Only one sub-micromolar example was identified in the primary screen. The hit A0009 was repurchased and retesting the compound confirmed the data obtained from the screening stocks. A0009 was tested in the kinase panel but did not hit any of the 76 kinases, A0009 is also a >75 fold selective inhibitor of *P. falciparum* over MRC5 cell growth. MMV was already developing analogues of MMV09 so no further work was conducted on this series. Table 3 (main body of the paper) contains a summary of A0009 and its physical properties as generated by Stardrop.

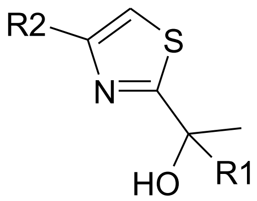

Figure S4 – MMV09 Scaffold

MMV11

MMV11 is a 3,6-disubstituted imidazo[1,2-b]pyridazine (Supplementary Figure 5). Only one sub-micromolar example of this series was identified in the primary screen. A0011 was tested in the kinase panel inhibiting RSK1, RSK2, Aurora B and Lck by between 50-80% at 10 µM. A0011 is only a 10 fold selective inhibitor of *P. falciparum* over MRC5 cell growth. The hit A0011 could not be repurchased and as this series was under development by MMV, no further work was conducted. Table 3 (main body of the paper) contains a summary of A0011 and its physical properties as generated by Stardrop.

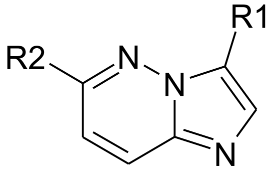

Figure S5 – MMV11 Scaffold

Chemistry Experimental

Chemistry. *General***.** Solvents and reagents were purchased from commercial suppliers and used without further purification. Dry solvents were purchased in sure sealed bottles stored over molecular sieves. Normal phase TLCs were carried out on pre-coated silica plates (Kieselgel 60 F_{254}, BDH) with visualisation *via* UV light (UV254/365 nm) and/or ninhydrin solution. Flash chromatography was performed using Combiflash Companion Rf (Teledyne ISCO) and prepacked RediSep silica gel columns purchased from Teledyne ISCO. Preparative HPLC separations were performed with a Gilson HPLC (321 pumps, 819 injection module, 215 liquid handler/injector) connected to a Gilson 155 UV/vis detector. HPLC chromatographic separations were conducted using Waters XBridge C18 columns, 19 x 100 mm, 5 um particle size; using 0.1% ammonia in water or 0.1% formic acid in water (solvent A) and acetonitrile (solvent B) as mobile phase. ^{1}H NMR spectra were recorded on a Bruker Avance DPX 500 spectrometer (^{1}H at 500.1 MHz). Chemical shifts (δ) are expressed in ppm recorded using the residual solvent as the internal reference in all cases. Signal splitting patterns are described as singlet (s), doublet (d), triplet (t), quartet (q), multiplet (m), broadened (br), or a combination thereof. Coupling constants (*J*) are quoted to the nearest 0.1 Hz. Low resolution electrospray (ES) mass spectra were recorded on a Bruker Daltonics MicroTof mass spectrometer, run in positive mode. LC-MS analysis and chromatographic separation were conducted with a Brucker Daltonics MicrOTOf mass spectrometer or an Agilent Technologies 1200 series HPLC connected to an Agilent Technologies 6130 quadrupole LC/MS, where both instruments were connected to an Agilent diode array detector. The column used was a Waters XBridge column (50 mm × 2.1 mm, 3.5 µm particle size,) and the compounds were eluted with a gradient of 5 to 95% acetonitrile/water +0.1% Ammonia. All final compounds showed chemical purity ≥ 95% as determined by the UV chromatogram (190-450nm) obtained by LC-MS analysis. Unless otherwise stated herein reactions have not been optimized. Compounds **A0012, A0036, A0037** and **A0038** were purchased from Enamine and tested without further purification after purity was determined to be ≥ 95% by integration of the UV chromatogram (190-450nm) obtained by LC-MS analysis.

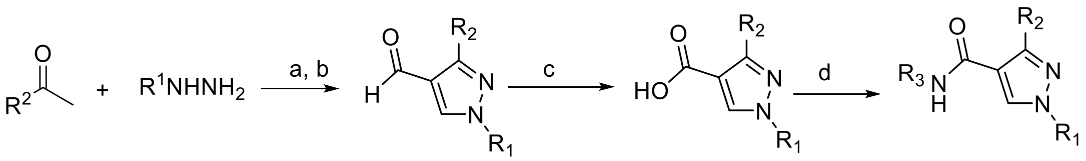

Conditions: (a) AcOH, EtOH, 90°C, 1h 30min; (b) $POCl_3$, DMF, reflux, overnight; (c) $KMnO_4$, KOH, dioxane/water 4/1; (d) Amine, EDC, HOBt, DIPEA, DMF

Supplementary Figure 6. General synthetic route.

General procedure A: Synthesis of pyrazole-4-aldehydes. To a solution of the methyl ketone (1 eq) and the corresponding hydrazine (1 eq) in ethanol (1 mL/mmol), acetic acid (1 mL/ 25 mmol) was added dropwise at room temperature. Reaction mixture was heated at 90°C for 1h and 30 min. Solvents were removed under reduced pressured and residue partitioned between NaHCO3 saturated aqueous solution (50 mL) and DCM (250 mL). The organic layer was dried over $MgSO_4$ and solvents were removed under reduced pressured. The residue was dissolved in DMF (7 mL) and added to a previously prepared mixture of $POCl_3$ (2 eq) and DMF (2 eq) dropwise in an ice bath. The mixture was stirred at room temperature for 30 min and then refluxed overnight. Reaction was quenched over ice water (50 mL) followed by 2.5 M NaOH (15 mL) and extracted with DCM (250 mL). The organic layer was dried over $MgSO_4$ and solvents were removed under reduced pressured to obtain the desire product that was used without further purification.

General procedure B: Oxidation of pyrazole-4-aldehydes. To a solution of the pyrazole-4-carbaldehyde (1 eq) in a mixture of dioxane/water 4/1 (5 mL/mmol), a solution of KOH (1.5 eq) in water (8 mL) and $KMnO_4$ (1.5 eq) were added. The reaction mixture was stirred overnight at room temperature. The reaction was quenched with a mixture of hydrogen peroxide/water 1/1 (10 mL) and filtered through Celite. Solvents were removed and pH was adjusted to pH 3 with 3N HCl. Precipitate was filtered and washed with water. The product was dried under vacuum and used without further purification.

General procedure C: Synthesis of amides. To a solution of the pyrazoloze-4-carboxylic acid (1 eq) in DMF (5 mL), the corresponding amine (1.2 eq), diisopropylethylamine (1.2 eq), EDC (2 eq) and HOBt (2 eq) were added at room temperature. The reaction mixture was stirred overnight at room temperature. The reaction was partitioned between $NaHCO_3$ saturated aqueous solution (10 mL)

and DCM (2 x 25 mL). The combined organic layers were dried over $MgSO_4$ and solvents were removed under reduced pressure. The product was purified by column chromatography.

1-Phenyl-3-(thiophen-2-yl)-1*H*-pyrazole-4-carboxylic acid. Prepared following general procedures A and B starting from 1 2-acetylthiophene (1.7 mL, 16 mmol) and phenyl hydrazine (1.6 mL, 16 mmol) to obtain 1-phenyl-3-(thiophen-2-yl)-1*H*-pyrazole-4-carboxylic acid as a brown solid (1.3 g, 30% yield). ^{1}H NMR (500 MHz, DMSO) δ 9.08 (s, 1H), 8.17 (d, J=2.7 Hz, 1H), 7.96 (d, J=7.7 Hz, 2H), 7.62 - 7.59 (m, 1H), 7.55 (dd, J=8.0, 8.0 Hz, 2H), 7.40 (dd, J=7.4, 7.4 Hz, 1H), 7.17 (dd, J=3.8, 5.0 Hz, 1H). LC-MS (ESI) *m/z* 271 $[M + H]^+$.

***N*-(1H-benzo[d]imidazol-2-yl)-1-phenyl-3-(thiophen-2-yl)-1*H*-pyrazole-4-carboxamide (A0034).** Prepared following general procedure C starting from 1-phenyl-3-(thiophen-2-yl)-1*H*-pyrazole-4-carboxylic acid (200 mg, 0.7 mmol) and purified by column chromatography using a silica cartridge (12 g) and DCM (A) and 10% MeOH in DCM (B) as eluent to obtain *N*-(1H-benzo[d]imidazol-2-yl)-1-phenyl-3-(thiophen-2-yl)-1*H*-pyrazole-4-carboxamide as a white solid (13 mg, 5% yield). ^{1}H NMR (500 MHz, DMSO) δ 9.32 (s, 1H), 8.19 (d, J=3.0 Hz, 1H), 7.90 (d, J=7.9 Hz, 2H), 7.66 - 7.59 (m, 3H), 7.49 (dd, J=3.2, 5.4 Hz, 2H), 7.47 - 7.41 (m, 2H), 7.20 - 7.11 (m, 4H). LC-MS (ESI) *m/z* 386 ($[M + H]^+$.

3-Isopropyl-1-phenyl-1*H*-pyrazole-4-carbaldehyde. Prepared following general procedure A starting from 3-methyl-2-butanone (2.2 mL, 20 mmol) and phenyl hydrazine (1 eq) to obtain 3-isopropyl-1-phenyl-1H-pyrazole-4-carbaldehyde as a brown solid (0.4 g, 10% yield). ^{1}H NMR (500 MHz, $CDCl_3$) δ 10.05 (s, 1H), 8.37 (s, 1H), 7.75 - 7.72 (m, 2H), 7.52 - 7.48 (m, 2H), 7.37 (t, J=7.4 Hz, 1H), 3.56 - 3.47 (m, 1H), 1.43 - 1.42 (m, 6H). LC-MS (ESI) *m/z* 215 $[M + H]^+$.

3-Isopropyl-1-phenyl-1*H*-pyrazole-4-carboxylic acid. Prepared following general procedure B starting from 3-isopropyl-1-phenyl-1H-pyrazole-4-carbaldehyde (0.4 g, 2 mmol) to obtain 3-isopropyl-1-phenyl-1*H*-pyrazole-4-carboxylic acid as a brown solid (0.4 g, 84% yield). ^{1}H NMR (500 MHz, DMSO) δ 12.44 - 12.44 (m, 1H), 8.87 (s, 1H), 7.89 (d, J=7.6 Hz, 2H), 7.53 - 7.49 (m, 2H), 7.34 (t, J=7.4 Hz, 1H), 3.58 - 3.51 (m, 1H), 1.29 (d, J=6.9 Hz, 6H). LC-MS (ESI) *m/z* 231 $[M + H]^+$.

3-Isopropyl-1-phenyl-*N*-(pyridin-3-yl)-1*H*-pyrazole-4-carboxamide (A0039). Prepared following general procedure C starting from 3-isopropyl-1-phenyl-1*H*-pyrazole-4-carboxylic acid (158 mg, 0.7

mmol) and purified by column chromatography using a silica cartridge (12 g) and DCM (A) and 10% NH_3-MeOH in DCM (B) as eluent to obtain 3-isopropyl-1-phenyl-N-(pyridin-3-yl)-1*H*-pyrazole-4-carboxamide as a white solid (149 mg, 70% yield). ^{1}H NMR (500 MHz, DMSO) δ 10.07 (s, 1H), 9.07 (s, 1H), 8.86 (d, J=2.2 Hz, 1H), 8.30 (dd, J=1.4, 4.7 Hz, 1H), 8.17 - 8.14 (m, 1H), 7.83 (d, J=7.6 Hz, 2H), 7.59 - 7.55 (m, 2H), 7.41 - 7.36 (m, 2H), 3.65 - 3.56 (m, 1H), 1.32 (d, J=6.9 Hz, 6H). LC-MS (ESI) *m/z* 307 $[M + H]^+$.

3-Methyl-1-phenyl-1*H*-pyrazole-4-carbaldehyde. Prepared following general procedure A starting from acetone (1.5 mL, 20 mmol) and phenyl hydrazine (1 eq) to obtain 3-methyl-1-phenyl-1H-pyrazole-4-carbaldehyde as a brown solid (1.3 g, 35 % yield). ^{1}H NMR (500 MHz, $CDCl_3$) δ 10.00 (s, 1H), 8.35 (s, 1H), 7.71 - 7.68 (m, 2H), 7.51 - 7.47 (m, 2H), 7.37 (dd, J=7.4, 7.4 Hz, 1H), 2.59 (s, 3H). LC-MS (ESI) *m/z* 187 $[M + H]^+$.

3-Methyl-1-phenyl-1*H*-pyrazole-4-carboxylic acid. Prepared following general procedure B starting from 3-methyl-1-phenyl-1H-pyrazole-4-carbaldehyde (1.4 g, 7.2 mmol) to obtain 3-methyl-1-phenyl-1*H*-pyrazole-4-carboxylic acid as a brown solid (0.8 g, 56% yield). ^{1}H NMR (500 MHz, CDCl3) δ 8.45 (s, 1H), 7.72 (d, J=7.4 Hz, 2H), 7.52 - 7.49 (m, 2H), 7.37 (t, J=7.4 Hz, 1H), 2.62 (s, 3H). LC-MS (ESI) *m/z* 203 $[M + H]^+$.

3-Methyl-1-phenyl-*N*-(pyridin-3-yl)-1*H*-pyrazole-4-carboxamide (A0040). Prepared following general procedure C starting from 3-methyl-1-phenyl-1*H*-pyrazole-4-carboxylic acid (139 mg, 0.7 mmol) and purified by column chromatography using a silica cartridge (12 g) and DCM (A) and 10% NH_3-MeOH in DCM (B) as eluent to obtain 3-methyl-1-phenyl-*N*-(pyridin-3-yl)-1*H*-pyrazole-4-carboxamide as a white solid (78 mg, 40% yield). ^{1}H NMR (500 MHz, MeOD) δ 10.86 (s, 1H), 9.90 (s, 1H), 9.68 (d, J=2.4 Hz, 1H), 9.11 (dd, J=1.3, 4.7 Hz, 1H), 8.96 (A, J=1.5, 2.6, 8.3 Hz, 1H), 8.63 (d, J=7.6 Hz, 2H), 8.40 - 8.36 (m, 2H), 8.23 - 8.18 (m, 2H), 3.31 (s, 3H). LC-MS (ESI) *m/z* 279 $[M + H]^+$.

4-(4-Formyl-3-(pyridin-3-yl)-1*H*-pyrazol-1-yl)benzonitrile. Prepared following general procedure A starting from 2-acetylpyridine (1.8 mL, 16 mmol) and 4-cyanophenyl hydrazine hydrochloride (1 eq) to obtain 4-(4-formyl-3-(pyridin-3-yl)-1*H*-pyrazol-1-yl)benzonitrile as an off-white solid (2.4 g, 55 % yield). ^{1}H NMR (500 MHz, DMSO) δ 10.02 (s, 1H), 9.59 (s, 1H), 9.12 (d, J=1.4 Hz, 1H), 8.70 (dd, J=1.7,

4.8 Hz, 1H), 8.37 - 8.33 (m, 1H), 8.24 (d, J=8.8 Hz, 2H), 8.10 (d, J=8.8 Hz, 2H), 7.57 (dd, J=5.2, 7.6 Hz, 1H). LC-MS (ESI) *m/z* 275 $[M + H]^+$.

1-(4-Cyanophenyl)-3-(pyridin-3-yl)-1*H*-pyrazole-4-carboxylic acid. Prepared following general procedure B starting from 4-(4-formyl-3-(pyridin-3-yl)-1*H*-pyrazol-1-yl)benzonitrile (2.4 g, 8.7 mmol) to obtain 1-(4-cyanophenyl)-3-(pyridin-3-yl)-1*H*-pyrazole-4-carboxylic acid as an off-white solid (2.1 g, 85% yield). ^{1}H NMR (500 MHz, DMSO) δ 9.39 (s, 1H), 9.35 (s, 1H), 8.91 (d, J=4.4 Hz, 1H), 8.86 (d, J=8.2 Hz, 1H), 8.25 (d, J=9.0 Hz, 2H), 8.07 - 8.00 (m, 3H).LC-MS (ESI) *m/z* 291 $[M + H]^+$.

***N*-(1H-Benzo[d]imidazol-2-yl)-1-(4-cyanophenyl)-3-(pyridin-3-yl)-1*H*-pyrazole-4-carboxamide (A0041).** Prepared following general procedure C starting from 1-(4-cyanophenyl)-3-(pyridin-3-yl)-1*H*-pyrazole-4-carboxylic acid (199 mg, 0.7 mmol) and purified by column chromatography using a silica cartridge (4 g) and DCM (A) and 20% NH_3-MeOH in DCM (B) as eluent to obtain *N*-(1H-benzo[d]imidazol-2-yl)-1-(4-cyanophenyl)-3-(pyridin-3-yl)-1*H*-pyrazole-4-carboxamide as a white solid (60 mg, 21% yield). ^{1}H NMR (500 MHz, DMSO) δ 9.49 (s, 1H), 9.05 (d, J=2.2 Hz, 1H), 8.65 (dd, J=1.7, 4.8 Hz, 1H), 8.29-8.26 (m, 1H), 8.15 (d, J=8.9 Hz, 2H), 8.11 (d, J=8.9 Hz, 2H), 7.54-7.51 (m, 1H), 7.48-7.44 (m, 2H), 7.14-7.11 (m, 2H). LC-MS (ESI) *m/z* 406 $[M + H]^+$.

1-(4-Fluorophenyl)-3-(pyridin-3-yl)-1*H*-pyrazole-4-carbaldehyde. Prepared following general procedure A starting from 2-acetylpyridine (1.8 mL, 16 mmol) and 4-fluorophenyl hydrazine hydrochloride (1 eq) to obtain 1-(4-fluorophenyl)-3-(pyridin-3-yl)-1*H*-pyrazole-4-carbaldehyde as an off-white solid (1.3 g, 36 % yield). ^{1}H NMR (500 MHz, $CDCl_3$) δ 10.08 (s, 1H), 9.13 (d, J=1.4 Hz, 1H), 8.74 (dd, J=1.7, 4.9 Hz, 1H), 8.54 (s, 1H), 8.26 - 8.23 (m, 1H), 7.82 - 7.79 (m, 2H), 7.47 (dd, J=4.9, 7.1 Hz, 1H), 7.29 - 7.24 (m, 2H). LC-MS (ESI) *m/z* 268 $[M + H]^+$.

1-(4-Fluorophenyl)-3-(pyridin-3-yl)-1*H*-pyrazole-4-carboxylic acid. Prepared following general procedure B starting from 1-(4-fluorophenyl)-3-(pyridin-3-yl)-1*H*-pyrazole-4-carbaldehyde (1.3 g, 4.9 mmol) to obtain 1-(4-fluorophenyl)-3-(pyridin-3-yl)-1*H*-pyrazole-4-carboxylic acid as an off-white solid (1.3 g, 96% yield). ^{1}H NMR (500 MHz, DMSO) δ 9.26 (s, 1H), 9.19 (s, 1H), 8.84 (d, J=1.6 Hz, 1H), 8.73 (d, J=8.0 Hz, 1H), 8.08 - 8.04 (m, 2H), 7.92 (dd, J=5.4, 7.8 Hz, 1H), 7.42 (dd, J=8.8, 8.8 Hz, 2H). LC-MS (ESI) *m/z* 284 $[M + H]^+$.

***N*-(1H-Benzo[*d*]imidazol-2-yl)-1-(4-fluorophenyl)-3-(pyridin-3-yl)-1*H*-pyrazole-4-carboxamide (A0042).** Prepared following general procedure C starting from 1-(4-fluorophenyl)-3-(pyridin-3-yl)-1*H*-pyrazole-4-carboxylic acid (195mg, 0.7 mmol) and purified by column chromatography using a silica cartridge (4 g) and DCM (A) and 20% NH_3-MeOH in DCM (B) as eluent to obtain *N*-(1H-benzo[*d*]imidazol-2-yl)-1-(4-fluorophenyl)-3-(pyridin-3-yl)-1*H*-pyrazole-4-carboxamide as an off-white solid (18 mg, 7% yield). ^{1}H NMR (500 MHz, MeOD) δ 9.03 (d, J=1.4 Hz, 1H), 8.87 (s, 1H), 8.48 (d, J=3.6 Hz, 1H), 8.33 (d, J=8.0 Hz, 1H), 7.84 - 7.80 (m, 2H), 7.47 - 7.42 (m, 3H), 7.25 (dd, J=8.7, 8.7 Hz, 2H), 7.19 (dd, J=3.1, 5.9 Hz, 2H). LC-MS (ESI) *m/z* 399 $[M + H]^+$.

1-(*tert*-Butyl)-3-(pyridin-3-yl)-1*H*-pyrazole-4-carbaldehyde. Prepared following general procedure A starting from 2-acetylpyridine (1.8 mL, 16 mmol) and *tert*-butyl hydrazine hydrochloride (1 eq) to obtain 1-(*tert*-butyl)-3-(pyridin-3-yl)-1*H*-pyrazole-4-carbaldehyde as an off-white solid (1.9 g, 49 % yield). ^{1}H NMR (500 MHz, $CDCl_3$) δ 9.97 (s, 1H), 9.06 (d, J=1.4 Hz, 1H), 8.68 (dd, J=1.3, 4.8 Hz, 1H), 8.20 - 8.18 (m, 2H), 7.42 (dd, J=5.0, 7.7 Hz, 1H), 1.69 (s, 9H). LC-MS (ESI) *m/z* 230 $[M + H]^+$.

1-(*tert*-Butyl)-3-(pyridin-3-yl)-1*H*-pyrazole-4-carboxylic acid. Prepared following general procedure B starting from 1-(*tert*-butyl)-3-(pyridin-3-yl)-1H-pyrazole-4-carbaldehyde (1.9 g, 8.1 mmol) to obtain 1-(*tert*-butyl)-3-(pyridin-3-yl)-1*H*-pyrazole-4-carboxylic acid as an off-white solid (1.2 g, 60% yield). ^{1}H NMR (500 MHz, DMSO) δ 12.46 - 12.45 (m, 1H), 8.91 (d, J=1.4 Hz, 1H), 8.56 (dd, J=1.4, 4.7 Hz, 1H), 8.39 (s, 1H), 8.15 - 8.11 (m, 1H), 7.44 (dd, J=5.0, 7.6 Hz, 1H), 1.59 (s, 9H). LC-MS (ESI) *m/z* 246 $[M + H]^+$.

***N*-(1H-Benzo[*d*]imidazol-2-yl)-1-(*tert*-butyl)-3-(pyridin-3-yl)-1*H*-pyrazole-4-carboxamide (A0043).** Prepared following general procedure C starting from 1-(tert-butyl)-3-(pyridin-3-yl)-1*H*-pyrazole-4-carboxylic acid (169 mg, 0.7 mmol) and purified by column chromatography using a silica cartridge (4 g) and DCM (A) and 20% NH_3-MeOH in DCM (B) as eluent to obtain *N*-(1H-benzo[*d*]imidazol-2-yl)-1-(tert-butyl)-3-(pyridin-3-yl)-1*H*-pyrazole-4-carboxamide as a white solid (37 mg, 15% yield. ^{1}H NMR (500 MHz, MeOD) δ 8.96 (d, J=1.6 Hz, 1H), 8.54 (s, 1H), 8.51 (dd, J=1.6, 4.9 Hz, 1H), 8.28 - 8.24 (m, 1H), 7.46 (dd, J=4.9, 7.9 Hz, 1H), 7.41 - 7.38 (m, 2H), 7.18 - 7.16 (m, 2H), 1.64 (s, 9H). LC-MS (ESI) *m/z* 361 $[M + H]^+$.

1-Methyl-3-(thiophen-2-yl)-1*H*-pyrazole-4-carbaldehyde. Prepared following general procedure A starting from 2-acetylthiophene (1.7 mL, 16 mmol) and methyl hydrazine (1 eq) to obtain 1-methyl-3-(thiophen-2-yl)-1*H*-pyrazole-4-carbaldehyde as a brown oil (2.4 g, 79% yield). ^{1}H NMR (500 MHz, $CDCl_3$) δ 10.02 (s, 1H), 7.95 (s, 1H), 7.78 (td, J=1.0, 3.6 Hz, 1H), 7.37 (td, J=1.0, 5.1 Hz, 1H), 7.13-7.11 (m, 1H), 3.96 (s, 3H). LC-MS (ESI) *m/z* 193 $[M + H]^+$.

1-Methyl-3-(thiophen-2-yl)-1*H*-pyrazole-4-carboxylic acid. Prepared following general procedure B starting from 1-methyl-3-(thiophen-2-yl)-1*H*-pyrazole-4-carbaldehyde (2.4 g, 13 mmol) to obtain 1-methyl-3-(thiophen-2-yl)-1*H*-pyrazole-4-carboxylic acid as a yellow solid (1.2 g, 48% yield). ^{1}H NMR (500 MHz, DMSO) δ 12.43 - 12.43 (m, 1H), 8.30 (s, 1H), 8.08 (dd, J=1.1, 3.6 Hz, 1H), 7.51 (dd, J=1.1, 5.2 Hz, 1H), 7.10 (dd, J=3.7, 5.1 Hz, 1H), 3.88 (s, 3H). LC-MS (ESI) *m/z* 209 $[M + H]^+$.

1-Methyl-*N*-(pyridin-3-yl)-3-(thiophen-2-yl)-1*H*-pyrazole-4-carboxamide (A0044). Prepared following general procedure C starting from 1-methyl-3-(thiophen-2-yl)-1*H*-pyrazole-4-carboxylic acid (143 mg, 0.7 mmol) and purified by column chromatography using a silica cartridge (12 g) and DCM (A) and 10% MeOH in DCM (B) as eluent to obtain as a white solid (43 mg, xx% yield). ^{1}H NMR (500 MHz, $CDCl_3$) δ 8.36 (d, J=1.6 Hz, 1H), 8.30 (d, J=4.3 Hz, 1H), 8.13 - 8.08 (m, 2H), 7.98 (s, 1H), 7.52 - 7.48 (m, 2H), 7.18 (dd, J=3.6, 5.0 Hz, 1H), 3.92 (s, 3H). LC-MS (ESI) *m/z* 285 $[M + H]^+$.
